# The use of EQ-5D-5L as a patient-reported outcome measure in evaluating community rehabilitation services in Alberta, Canada

**DOI:** 10.1186/s12955-023-02207-w

**Published:** 2023-11-17

**Authors:** Hilary Short, Fatima Al Sayah, Katie Churchill, Eileen Keogh, Lisa Warner, Arto Ohinmaa, Jeffrey A. Johnson

**Affiliations:** 1 School of Public Health, University of Alberta, Edmonton, AB T6G 2E1 Canada; 2 Department of Rehabilitation Medicine, Allied Health Professional Practice and Education, Alberta Health Services, Edmonton, AB Canada; 3 Allied Health Professional Practice and Education, Alberta Health Services, Suite 300, 10216 – 124 Street, Edmonton, AB T5N 4A3 Canada; 4 Allied Health Professional Practice and Education, Alberta Health Services, Calgary, AB Canada

**Keywords:** Patient-reported outcome measures, EQ-5D, Health-related quality of life, Rehabilitation

## Abstract

**Background:**

The purpose of this study was to describe the characteristics and health-related quality of life (HRQL) of patients accessing community rehabilitation services in Alberta, Canada, using routinely collected EQ-5D-5L data, and explore factors associated with the impact of these services.

**Methods:**

A retrospective, longitudinal, observational design was used. Patients completed the EQ-5D-5L and demographic questions at intake and end of rehabilitation care. Change in EQ-5D-5L dimensions from intake until end of rehabilitation was examined using the Pareto Classification of Health Change. Change scores were calculated for the EQ-5D-5L index, VAS, and total sum scores. Change groups in the EQ-5D-5L index and VAS scores, were defined by minimally important differences of 0.04 and 7.0, respectively. One level change was considered important for the total sum score. Effect size of the change in index, VAS, and total sum scores was also examined. Chi-squared tests were conducted to examine whether change in EQ-5D-5L varied by age, gender, region, and having anxiety/depression at intake.

**Results:**

Three service programs were examined; pulmonary rehabilitation (*n* = 542), group-based community exercise (*n* = 463), and physiotherapy for bone and joint care (*n* = 391). At intake, HRQL in all programs was lower than that of the general Alberta population norms and improved by end of rehabilitation. The mean (SD) change in index, VAS, and total sum scores were 0.02 (0.13), 6.0 (18.3), and − 0.5 (2.4) in pulmonary rehabilitation, 0.06 (0.13), 6.6 (18.7), − 1.2 (2.4) in community exercise, and 0.13 (0.16), 1.2 (0.9), and − 2.8 (2.8) in physiotherapy, respectively. Based on change of the index score, 24% deteriorated, 38% improved, and 38% had no change in pulmonary rehabilitation; 17% deteriorated, 51% improved, and 32% had no change in community exercise; 5% deteriorated, 72% improved, and 23% had no change in physiotherapy. Similar trends were seen in the VAS and total sum scores. Older age, urban region, and having anxiety/depression at intake were associated with positive change in EQ-5D-5L.

**Conclusions:**

The results of this study are intended to inform program/service level decisions by describing the characteristics and HRQL of patients accessing community rehabilitation, as well as the predictors of change in health status, which will help direct future program growth and service changes.

## Background

Community rehabilitation is a person-centred approach that aims to maintain or improve patient functional abilities, prevent and deter illness and disability, and enhance health-related quality of life (HRQL) in an outpatient setting [1, 2]. Recently, there has been growing recognition of the importance of involving the patient perspective in evaluating clinical services in order to improve experience in rehabilitation care and other clinical settings [3, 4]. One method that health systems have adopted to achieve this is through the use of patient-reported outcome measures (PROMs). PROMs are tools used to measure patients’ direct perceptions regarding their health without others’ interpretations [5]. The use of PROMs have various roles in community rehabilitation services such as informing clinical practice, enhancing patient-centred care, supporting health services programming, directing performance measurement, and contributing to quality improvement [6–8]. However, routine, standardized use of PROMs has not been extensively adopted or reported in rehabilitation care [9], especially at the program or service level [7].

In the western Canadian province of Alberta, Alberta Health Services (AHS), responsible for province-wide provision of tertiary and community care, implemented a Rehabilitation Model of Care (R-MoC) to promote patient-centred care, provincial standardization, and data-driven service model [10, 11]. As part of the R-MoC, the EQ-5D-5L was adopted as a generic PROM and gradually implemented across community and outpatient rehabilitation services beginning in 2017. The process for selecting the EQ-5D-5L included stakeholder consultations, literature review, population needs assessment, current and future state reports, and a gap analysis [6]. Additionally, AHS had endorsed the EQ-5D-5L as the generic PROM for use in the healthcare system in 2015, and further embedding the ability to capture the EQ-5D-5L and other PROMs in *Connect Care*, the province-wide electronic patient medical information system [12]. Therefore, the EQ-5D-5L was chosen for use in the R-MoC as it could be embedded within workflows to facilitate patient-centred conversations; act as a quality indicator for programs and services; and inform health system planning and decision making [6]. An early adopter group of 18 sites with diverse service models and locations was identified to pilot the implementation of standardized routine outcome measurement from 2017 to 2018 [6]. In 2018, learnings from the pilot were used to spread and scale the R-MoC to an additional 152 adult community, outpatient rehabilitation sites across Alberta.

Very few studies have examined routinely collected PROMs in the care of rehabilitation patients and there is a significant gap in the literature on how such data can be used to inform local decision-makers on the performance of various programs or services and quality improvement initiatives. The aim of this study was to use routinely collected PROMs data (namely the EQ-5D-5L) to describe the characteristics and HRQL of patients accessing community rehabilitation services in Alberta, Canada, and explore factors associated with the impact of these services.

## Methods

### Data source

All patients participating in sites that were part of R-MoC implementation and data collection (*n* = 152 rehabilitation sites) were invited to complete a survey at intake (pre) and at the end (post) of receiving a rehabilitation service as part of routine outcome measurement. Survey content included the EQ-5D-5L, age, gender, and region where rehabilitation services were accessed (urban vs. rural). Included patients were 18 years or older and willing and able to complete the survey. Patients with cognitive impairment were excluded. Depending on the site, surveys were collected by iPad, laptop computer, or paper. All survey responses were stored in a secure electronic platform within the AHS firewall. A total of 2285 rehabilitation patients had data drawn from electronic medical records between December 1, 2018 and April 30, 2021. Only individuals with a complete pre and post EQ-5D-5L were included in the analysis. Records were excluded if they completed a pre and post EQ-5D-5L survey on the same day (*n* = 107). Of a total of 14 rehabilitation service types, we focused on three that had sufficient samples for analysis: pulmonary rehabilitation, group-based community exercise, and physiotherapy for bone and joint care.

Pulmonary rehabilitation programs provide rehabilitation assessment and treatment services to adults who have a chronic lung condition. The program consists of both group education and supervised exercise sessions to help people manage their lung diseases and improve their health and quality of life. The most common patient diagnosis in this group was chronic obstructive pulmonary disease and clinicians providing the service include respiratory therapists and physiotherapists.

Group-based community exercise programs consisted of a series of group education and supervised exercise sessions led by health professionals for persons with health conditions that impact their mobility and their ability to participate in home, work, or leisure activities. The program includes a variety of group exercise programs offered in a community setting with the aim of assisting participants with health conditions develop skills and confidence in self-directed physical activity in their home or community. The sessions occur over several weeks and include both exercise participation and education on the benefits of exercise. The clinicians involved in this program may include physiotherapists, kinesiologists, and occupational therapists.

Physiotherapy programs for bone and joint care provided treatment for individuals with specific physical concerns to help improve their function, their understanding of their condition, and what they can do to be healthy and independent, including services for general joint or muscle conditions or injuries, recent fractures or orthopedic surgeries, and recent hip or knee replacements. The clinical services were primarily provided by physiotherapists for lower extremity orthopaedic concerns (e.g., rehabilitation after hip or knee surgery or fracture). The goal of physical therapy services was to improve functional status and HRQL with exercise as a common treatment modality.

### Measures

The EQ-5D-5L is a generic preference-based measure of HRQL that includes five dimensions (mobility, self-care, usual activities, pain/discomfort, anxiety/depression), each with five levels of problems (1 = none, 2 = mild, 3 = moderate, 4 = severe, 5 = extreme), describing 3125 distinct health states [13, 14]. A preference-based summary score (i.e., an index score) is generated for each health state using a country-specific value set [15]. The Canadian EQ-5D-5L value set was applied to calculate the index score in this study [16]. The EQ-5D-5L also includes a visual analogue scale (VAS), which records the respondent’s self-rated health ‘today’ on a vertical, visual analogue scale, ranging from 0 “worst imaginable health state” to 100 “best imaginable health state” [14]. A total sum score, ranging from 5 to 25, can also be calculated from the EQ-5D-5L data by summing the levels on the five dimensions, whereby 5 represents the best health state (i.e., no problems in all five dimensions, 11111) and 25 represents the worst health state (i.e., severe problems in all five dimensions, 55555). The EQ-5D-5L also exhibits sound evidence of validity and reliability in similar patient populations [17–24]. Other variables collected included age group (18-24, 25-44, 45-64, 65-74, ≥75), gender, and urban vs. rural residence.

### Statistical analysis

General characteristics of the sample and changes in EQ-5D-5L from intake until the end of a rehabilitation intervention were examined in the three selected service programs (i.e., community exercise, pulmonary rehabilitation, physiotherapy). Approximate program intervention durations were determined based on the dates of completed pre and post EQ-5D-5L surveys, which may vary slightly than the dates of care delivered. Rehabilitation duration varies by program and individual. Change in the dimensions from intake until the end of rehabilitation was examined using the Pareto Classification of Health Change (PCHC) [25] as follows:

- Deteriorated: deterioration in the level of problems reported in one or more dimensions from intake to end of rehabilitation, with no improvement in other dimensions
- No change: level of problems reported (2-5 only) was the same at intake and end of rehabilitation
- Maintained perfect health: no problems reported (level 1) at intake and end of rehabilitation
- Improved: improvement in the level of problems reported in one or more dimensions from intake to end of rehabilitation, with no deterioration in other dimensions

The EQ-5D-5L index and VAS change scores (post minus pre) were calculated and assessed using the minimally important difference (MID) [26–28] as follows:

*EQ-5D-5L index score:*

- Deteriorated: change score ≤ − 0.04
- No change: − 0.04 < change score < 0.04
- Maintained perfect health: 0.95 (i.e., health state 11111) at intake and end of rehabilitation
- Improved: change score ≥ 0.04

*VAS Score:*

- Deteriorated: change score ≤ − 7.0
- No change: − 7.0 < change score < 7.0
- Maintained perfect health: 100 at intake and end of rehabilitation
- Improved: change score ≥ 7.0

No MID thresholds are available for the total sum score, as such, a change of one point in the total score was considered important and used to define change groups. Additionally, to assist in the interpretation of change in the EQ-5D-5L index, VAS, and total sum scores from pre to post, the effect size of the change was calculated by dividing the average change score by the standard deviation of the score at baseline. Effect size estimates were considered small (< 0.2), moderate (0.5) or large (> 0.8) [29].

The distribution of EQ-5D-5L levels of each dimension as well as mean (SD) index, VAS, and total sum scores were compared across service programs, participant factors (i.e., age, gender, region), and compared to Alberta general population norms [30]. Bivariate analyses were also conducted to examine the distribution of change in EQ-5D-5L index, VAS, and total sum scores (“deteriorated”, “no change”, “improved”) by age, gender, region, and the EQ-5D-5L anxiety/depression dimension at intake, using chi-squared tests. Region was excluded from bivariate analyses in the physiotherapy program as most of the sample was in one region. Due to limited “maintained perfect health” sample sizes within the index, VAS, and total sum scores, they were included in the “no change” group. The EQ-5D-5L anxiety/depression dimension level data was categorized into absent problems (level 1) and present problems (levels 2-5). A *p*-value < 0.05 was considered statistically significant. All statistical analyses were conducted in STATA 14.2 (Stata Corp LLC, College Station, Texas, USA).

## Results

### EQ-5D-5L in Pulmonary Rehabilitation

Among the 542 patients that received pulmonary rehabilitation services, half of the patients (59.2%) were older adults and half (50.9%) were male (Table 1). The primary conditions for presenting to pulmonary rehabilitation were chronic obstructive pulmonary disease (65.5%) or other respiratory condition (28.0%). The approximate duration of intervention (based on EQ-5D-5L measurement) ranged from 28 to 184 days, with a mean (SD) duration of 47.0 days (23.5) and median duration of 31.0 days.

**Table 1:** Demographics by sample and service area

Characteristic		Pulmonary Rehabilitation (*N* = 542)		Group-based Community Exercise (*N* = 463)		Physiotherapy (*N* = 391)	
		N	%	N	%	N	%
**Age**	18-24	0	0	6	1.30	9	2.30
	25-44	19	3.51	36	7.78	46	11.76
	45-64	184	33.95	153	33.05	130	33.25
	65-74	199	36.72	155	33.48	123	31.46
	≥75	122	22.51	107	23.11	72	18.41
	*Missing*	*18*	3.32	*6*	*1.30*	11	2.81
**Gender**	Male	276	50.92	129	27.86	154	39.39
	Female	247	45.57	329	71.06	237	60.61
	*Missing*	19	3.51	*5*	*1.08*	*0*	*0.0*
**Region**	Urban	489	90.22	221	47.73	10	2.56
	Rural	53	9.78	238	51.40	381	97.44
	*Missing*	*0*	*0*	*4*	*0.86*	*0*	*0*
**Primary Condition**	Chronic Obstructive Pulmonary Disease	355	65.50	0	0	0	0
	Other Respiratory Condition	152	28.04	0	0	0	0
	Chronic Pain	0	0	106	22.89	23	5.88
	Osteoarthritis or inflammatory arthritis	0	0	89	19.22	13	3.32
	Falls/Balance	0	0	41	8.86	0	0
	Neuromuscular/Neurological Diagnosis	0	0	29	6.26	0	0
	Depression/Anxiety or Psychosocial Condition	0	0	25	5.4	0	0
	Musculoskeletal Conditions (Sprains/Strains)	0	0	19	4.10	118	30.18
	Orthopedic Surgery	0	0	19	4.10	157	40.15
	Stroke	0	0	16	3.46	0	0
	Obesity	0	0	15	3.24	0	0
	Cardiovascular condition	0	0	13	2.81	0	0
	Diabetes	0	0	9	1.94	0	0
	Fracture	0	0	7	1.51	68	17.39
	Hypertension	0	0	5	1.08	0	0
	Other condition	0	0	24	5.18	11	2.81
	*Missing*	*35*	*6.46*	*46*	*9.94*	*2*	*0.51*

At intake, the proportion of patients reporting mild to extreme problems (levels 2-5) was 72.5% in mobility, 29.9% in self-care, 77.1% in usual activities, 70.5% in pain/discomfort, and 61.4% in anxiety/depression. The mean (SD) index score was 0.73 (0.17), the mean VAS score was 62.3 (19.4), and the mean total sum score was 10.2 (3.2) (Table 2).

**Table 2:** EQ-5D-5L dimensions, index, VAS, and total sum scores at intake and end of rehabilitation across the three programs compared to Alberta Norms

EQ-5D-5L		Pulmonary Rehabilitation (*N* = 542)		Group-based Community Exercise (*N* = 463)		Physiotherapy (*N* = 391)		^a^Alberta Norms Overall (*n* = 30,576)	^a^Alberta Norms 45+ years old (*n* = 19,003)
Dimension	Level	Intake	End of Rehab	Intake	End of Rehab	Intake	End of Rehab		
Mobility, %	1	27.49	32.47	20.95	33.48	32.74	58.82	72.8	63.7
	2	26.57	30.07	28.73	35.85	28.64	27.11	15.2	19.3
	3	35.42	30.44	39.52	23.54	28.39	12.53	8.5	11.9
	4	10.33	6.64	9.29	6.70	8.18	1.28	3.0	4.4
	5	0.18	0.37	1.51	0.43	2.05	0.26	0.5	0.7
Self-Care, %	1	70.11	69.37	66.52	72.79	60.10	85.42	94.1	92.0
	2	17.71	17.90	23.76	19.87	26.09	12.53	3.7	4.9
	3	10.52	10.89	8.21	6.70	12.28	1.79	1.8	2.5
	4	1.29	1.66	1.08	0.43	0.77	0.00	0.3	0.4
	5	0.37	0.18	0.43	0.22	0.77	0.26	0.2	0.2
Usual Activities, %	1	22.88	27.31	20.73	34.77	16.62	46.80	74.0	67.4
	2	32.10	37.08	35.21	34.34	30.18	38.36	15.3	18.4
	3	34.50	27.68	34.34	24.62	35.81	11.51	8.0	10.6
	4	9.23	7.20	8.64	5.18	10.23	2.81	1.8	2.4
	5	1.29	0.74	1.08	1.08	7.16	0.51	0.9	1.2
Pain / Discomfort, %	1	29.52	28.78	10.15	16.85	3.84	22.25	36.0	27.3
	2	30.63	35.79	31.97	40.17	33.76	55.75	38.8	41.0
	3	31.92	28.41	43.63	35.21	50.90	19.69	19.4	24.0
	4	7.56	6.64	12.53	7.13	11.00	2.30	4.5	6.0
	5	0.37	0.37	1.73	0.65	0.51	0.00	1.2	1.5
Anxiety / Depression, %	1	38.56	42.07	40.82	47.08	58.57	74.42	62.8	64.5
	2	33.58	33.03	32.61	33.48	27.37	17.39	23.4	22.4
	3	22.88	21.03	20.73	15.98	11.76	7.42	10.8	10.5
	4	4.06	3.69	4.75	2.59	2.05	0.26	1.9	1.7
	5	0.92	0.18	1.08	0.86	0.26	0.51	0.9	0.7
EQ-5D-5L index score, mean (SD)		0.73 (0.17)	0.75 (0.17)	0.70 (0.18)	0.76 (0.16)	0.71 (0.17)	0.84 (0.11)	0.84 (0.14)	0.82 (0.15)
VAS score, mean (SD)		62.3 (19.4)	68.3 (17.7)	65.3 (18.2)	71.8 (17.6)	68.2 (18.9)	80.4 (14.0)	77.4 (17.1)	75.8 (18.0)
EQ-5D-5L total sum score, mean (SD)		10.2 (3.2)	9.8 (3.2)	10.8 (3.1)	9.5 (3.0)	10.6 (2.9)	7.8 (2.4)	NR	NR

*NR* Not reported

^a^APERSU (2018). Alberta Population Norms for EQ-5D-5L

https://sites.google.com/ualberta.ca/apersu/about-eq-5d/eq-5d-population-norms?authuser=0

By the end of the rehabilitation intervention, there was an 1.3% decrease in the proportion of patients reporting no problems on all EQ-5D-5L dimensions, and the proportion of patients reporting problems in self-care and pain/discomfort increased slightly. Based on PCHC, 9.4% of patients had no change in their health status, 37.1% had an improvement, 23.4% had a deterioration, and 30.1% had a mixed change. By the end of the care intervention, there was an average increase of 0.02 (SD 0.12) in the index score (effect size = 0.1), an increase of 6.0 points (SD 18.3) in the VAS (effect size = 0.31), and a decrease of 0.45 (SD 2.35) in the total sum score (effect size = 0.13). All changes were of small magnitude, and those of the index and VAS scores did not reach MID thresholds.

**Fig. 1:**
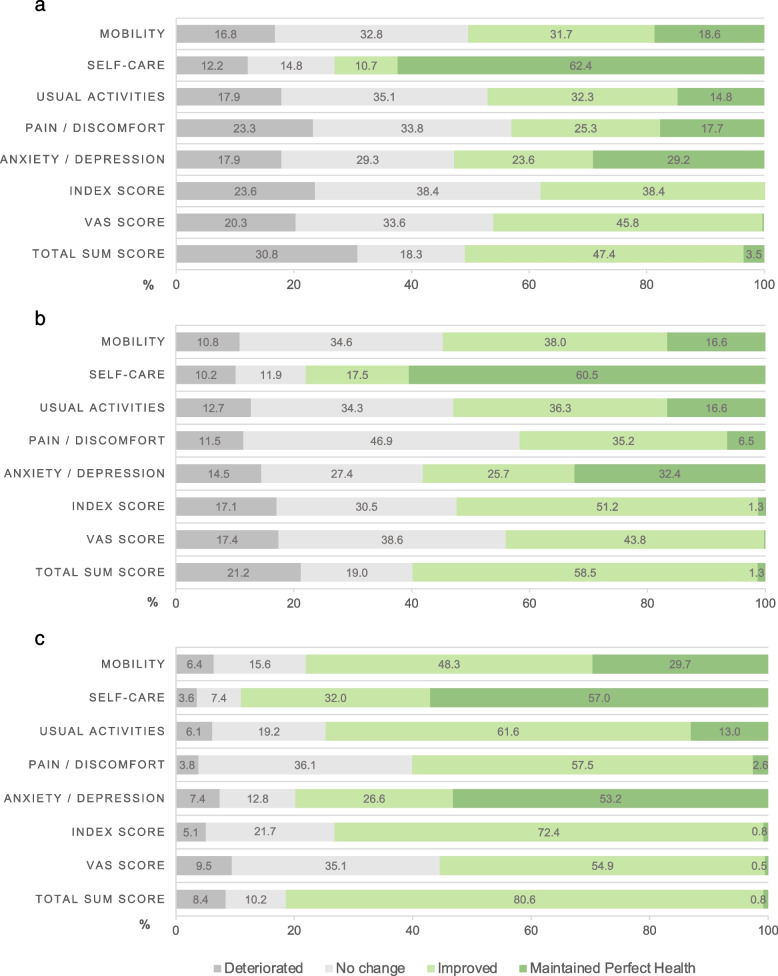
Change (%) in EQ-5D-5L dimensions, index, VAS, and total sum scores from intake until end of rehabilitation by program. **a** Pulmonary Rehabilitation. **b** Group-based Community Exercise. **c** Physiotherapy

The largest improvements were observed in usual activities followed by mobility. Less than half of patients improved based on the index (38.4%), VAS (45.8%), and total sum (47.4%) scores; health status either did not change or deteriorated in the other half (Fig. 1a).

### EQ-5D-5L in Group-based Community Exercise

Among the 463 patients that participated in group-based community exercise services, half of the patients (56.6%) were older adults, and the majority (71.1%) were female (Table 1). The top three primary conditions for presenting to community exercise were chronic pain (22.9%), osteoarthritis or inflammatory arthritis (19.2%), and falls/balance (8.9%). The duration of intervention ranged from 2 to 275 days, with a mean (SD) duration of 103.7 days (51.4) and median duration of 91 days.

At intake, the proportion of patients reporting mild to extreme problems (levels 2-5) was 79.1% in mobility, 33.5% in self-care, 79.3% in usual activities, 89.9% in pain/discomfort, and 59.2% in anxiety/depression. The mean (SD) index score was 0.70 (0.18), the mean VAS score was 65.3 (18.2), and the mean total sum score was 10.8 (3.1) (Table 2).

By the end of rehabilitation intervention, there was a 4.3% increase in the proportion of patients reporting no problems on all EQ-5D-5L dimensions, and the proportion of patients reporting problems on all dimensions decreased. Based on PCHC, 11.0% of patients had no change in their health status, 50.3% had an improvement, 16.9% had a deterioration, and 21.8% had a mixed change. By the end of the intervention, there was an average increase of 0.06 (SD 0.13) in the EQ-5D-5L index score (effect size = 0.3), and an increase of 6.6 points (SD 18.7) in the VAS score (effect size = 0.4), and a decrease of 1.22 (2.39) in the EQ-5D-5L total sum score (effect size =0.4). These changes were of small to moderate magnitude, with only the index score reaching the MID threshold.

The largest improvements were observed in mobility followed by usual activities, and then pain/discomfort. About half of the patients improved based on the index (51.2%), VAS (43.8%), and total sum (58.5%) scores; health status either did not change or deteriorated in the other half of patients (Fig. 1b).

### EQ-5D-5L in Physiotherapy

Among the 391 patients that received physiotherapy services, more than half of the patients (64.7%) were middle aged (45-65 years) and over half (60.6%) were female (Table 1). The top three primary conditions for presenting to physiotherapy were orthopaedic surgery (40.2%), musculoskeletal conditions (30.2%), and fracture (17.4%). The duration of intervention ranged from 28 to 397 days, with a mean (SD) duration of 67.3 days (46.0) and median duration of 61 days.

At intake, the proportion of patients reporting mild to extreme problems (levels 2-5) was 67.3% in mobility, 39.9% in self-care, 83.4% in usual activities, 96.2% in pain/discomfort, and 41.4% in anxiety/depression. The mean (SD) index score was 0.71 (0.17), the mean VAS score was 68.3 (18.9), and the mean total sum score was 10.6 (2.9) (Table 2).

By end of rehabilitation, there was a 11.5% increase in the proportion of patients reporting no problems on all EQ-5D-5L dimensions and the proportion of patients reporting problems on all dimensions decreased. Based on PCHC, 5.1% of patients had no change in their health status, 73.2% had an improvement, 5.4% had a deterioration, and 16.4% had a mixed change. By the end of rehabilitation, there was an average increase of 0.13 (SD 0.16) in the EQ-5D-5L index score (effect size = 0.8), an increase of 12.2 points (SD 18.4) in the VAS score (effect size = 0.7) and a decrease of 2.81 (2.79) in the total sum score (effect size =1.0). All changes were of moderate to large magnitude, and those of the index and VAS scores reached MID thresholds.

The largest improvements were observed in usual activities, followed by pain/discomfort and then mobility. Most patients improved based on the index (72.4%), VAS (54.9%), and total sum scores (80.6%) (Fig. 1c).

### Rehabilitation patients compared to Alberta general population

In pulmonary rehabilitation and group-based community exercise service programs, self-reported health based on the EQ-5D-5L was much worse than that of the general Alberta population at intake, regardless of the age group (Table 2). Patients receiving these services reported more problems on all EQ-5D-5L dimensions and has lower index and VAS scores. Despite slight improvements by the end of the rehabilitation service, self-reported health remained much lower than that of the general Alberta population (overall and in relevant age groups). Similarly, patients receiving physiotherapy reported more problems on all EQ-5D-5L dimensions at intake and had lower index and VAS scores compared to the general Alberta population. However, physiotherapy patients had larger improvements than pulmonary rehabilitation and community exercise patients in all EQ-5D-5L dimensions, especially in anxiety/depression. Moreover, the average index and VAS scores of physiotherapy patients surpassed the overall and relevant age groups of the general Alberta population norms (Table 2).

### Distribution of self-reported health by service program

In the pulmonary rehabilitation program, the distribution across the health status change categories (i.e., “deteriorated”, “no change”, “improved”) based on the VAS was statistically significant with age (Table 3). Of the patients aged 25-44, 68% experienced no change and only 10% improved. A much greater proportion of improvement (42-50%) was seen in the older age groups (*p* = 0.014). The anxiety/depression dimension at intake was also statistically significant with the health status change categories based on the index (*p* < 0.001) and total sum scores (*p* < 0.001). Of the pulmonary rehabilitation patients that reported mild-extreme problems (levels 2-5) on the anxiety/depression dimension at intake, 46 and 55% improved on the index score and total sum score, respectively. Comparatively, patients that reported no problems (level 1) on the anxiety/depression dimension at intake, 48% experienced no change in index scores and 38% experienced a deterioration in total sum score.

**Table 3:** Change (%) in EQ-5D-5L index, VAS, and total sum scores by age, gender, region, anxiety/depression at intake across programs

**Pulmonary Rehabilitation**					
**Index score** (*n*=524), %		**Deteriorated** (*n*=124)	^**a**^**No Change** (*n*=202)	**Improved** (*n*=198)	***P*-value**
**Age**	25-44	26.3	42.1	31.6	0.294
	45-64	27.7	38.0	34.2	
	65-74	23.6	34.7	41.7	
	75+	17.2	45.1	37.7	
**Gender**	Female	23.9	36.8	39.3	0.720
	Male	23.2	40.2	36.6	
**Region**	Urban	23.3	38.5	38.2	0.790
	Rural	26.4	34.0	39.6	
**Anxiety / Depression**	No problems (level 1)	25.8	47.9	26.3	**<0.001**
	Problems (levels 2-5)	22.2	31.8	46.0	
**VAS** (*n*=523), %		**Deteriorated** (*n*=107)	^**a**^**No Change** (*n*=175)	**Improved** (*n*=241)	***P*-value**
**Age**	25-44	21.1	68.4	10.5	**0.014**
	45-64	22.3	28.8	48.9	
	65-74	18.2	32.3	49.5	
	75+	21.3	36.9	41.8	
**Gender**	Female	23.1	30.4	46.6	0.250
	Male	18.2	36.0	45.8	
**Region**	Urban	20.9	33.1	46.0	0.483
	Rural	15.4	40.4	44.2	
**Anxiety / Depression**	No problems (level 1)	22.5	33.0	44.5	0.613
	Problems (levels 2-5)	19.0	34.3	46.7	
**Total Sum Score** (*n*=524), %		Deteriorated (*n*=163)	^**a**^**No Change** (*n*=115)	**Improved** (*n*=246)	***P*-value**
**Age**	25-44	31.6	26.3	42.1	0.710
	45-64	35.3	21.7	42.9	
	65-74	30.2	20.6	49.3	
	75+	26.2	23.8	50.0	
**Gender**	Female	30.0	23.5	46.6	0.721
	Male	31.9	20.7	47.5	
**Region**	Urban	30.7	21.9	47.4	0.971
	Rural	32.1	20.8	47.2	
**Anxiety / Depression**	No problems (level 1)	38.3	26.8	34.9	**<0.001**
	Problems (levels 2-5)	26.1	18.6	55.3	
**Group-based community exercise**					
**Index score** (*n*=457), %		**Deteriorated** (*n*=79)	^**a**^**No Change** (*n*=147)	**Improved** (*n*=231)	***P*-value**
Age	18-24	0.0	50.0	50.0	0.661
	25-44	19.4	22.2	58.3	
	45-64	16.3	31.4	52.3	
	65-74	19.4	30.3	50.3	
	75+	15.9	38.3	45.8	
Gender	Female	17.9	30.7	51.4	0.516
	Male	14.7	35.7	49.6	
Region	Urban	13.1	31.7	55.2	0.072
	Rural	20.6	32.4	47.1	
Anxiety / Depression	No problems (level 1)	20.6	38.1	41.3	**0.002**
	Problems (levels 2-5)	14.6	27.4	58.0	
**VAS** (*n*=455), %		**Deteriorated** (*n*=80)	^**a**^**No Change** (*n*=174)	**Improved** (*n*=201)	***P*-value**
**Age**	18-24	16.7	50.0	33.3	0.828
	25-44	13.9	30.6	55.6	
	45-64	17.7	36.0	46.4	
	65-74	18.1	42.6	39.4	
	75+	18.1	37.1	44.8	
**Gender**	Female	17.4	39.0	43.6	0.999
	Male	17.2	39.1	43.8	
**Region**	Urban	14.2	37.0	48.9	0.063
	Rural	20.6	40.3	39.1	
**Anxiety / Depression**	No problems (level 1)	16.0	42.6	41.5	0.390
	Problems (levels 2-5)	18.3	36.3	45.4	
**Total Sum Score** (*n*=457), %		**Deteriorated** (*n*=98)	^**a**^**No Change** (*n*=94)	**Improved** (*n*=265)	***P*-value**
Age	18-24	16.7	33.3	50.0	0.879
	25-44	25.0	16.7	58.3	
	45-64	17.7	19.6	62.8	
	65-74	23.9	20.7	55.5	
	75+	22.4	22.4	55.1	
Gender	Female	21.9	19.8	58.4	0.737
	Male	19.4	22.5	58.1	
Region	Urban	15.8	19.0	65.2	**0.009**
	Rural	26.1	21.9	52.1	
Anxiety / Depression	No problems (level 1)	26.5	22.2	51.3	**0.022**
	Problems (levels 2-5)	17.5	19.0	63.5	
**Physiotherapy**					
**Index score** (*n*=380), %		**Deteriorated** (*n*=20)	^**a**^**No Change** (*n*=86)	**Improved** (*n*=274)	***P*-value**
**Age**	18-24	0.0	11.1	88.9	0.096
	25-44	2.2	17.4	80.4	
	45-64	6.2	24.6	69.2	
	65-74	2.4	27.6	69.9	
	75+	11.1	15.3	73.6	
**Gender**	Female	4.2	23.2	72.6	0.583
	Male	6.5	21.4	72.1	
Anxiety / Depression	No problems (level 1)	5.7	29.7	64.6	**<0.001**
	Problems (levels 2-5)	4.3	12.4	83.3	
**VAS** (*n*=377), %		**Deteriorated** (*n*=37)	^**a**^**No Change** (*n*=135)	**Improved** (*n*=205)	***P*-value**
**Age**	18-24	0.0	44.4	55.6	0.208
	25-44	8.7	47.8	43.5	
	45-64	10.8	36.2	53.1	
	65-74	7.4	38.0	54.6	
	75+	14.1	22.5	63.4	
**Gender**	Female	8.1	36.6	55.3	0.469
	Male	11.8	34.0	54.3	
**Anxiety / Depression**	No problems (level 1)	8.8	42.3	48.9	**0.004**
	Problems (levels 2-5)	10.6	26.1	63.4	
**Total Sum Score **(*n*=380), %		**Deteriorated** (*n*=32)	^**a**^**No Change** (*n*=42)	**Improved** (*n*=306)	***P*-value**
**Age**	18-24	11.1	0.0	88.9	0.839
	25-44	6.5	6.5	87.0	
	45-64	9.2	11.5	79.2	
	65-74	6.5	12.2	81.3	
	75+	11.1	12.5	76.4	
**Gender**	Female	6.8	12.2	81.0	0.236
	Male	11.0	9.1	79.9	
**Anxiety / Depression**	No problems (level 1)	10.5	14.4	75.1	**0.005**
	Problems (levels 2-5)	5.6	6.2	88.3	

^a^Includes the ‘maintained perfect health’ group due to small sample sizes

*p*<0.05

In group-based community exercise, the distribution across the total sum score change categories was statistically significant with region and anxiety/depression at intake (Table 3). Of those who lived in urban areas, 65% experienced an improvement on the total sum score, compared to 52% who lived in rural areas (*p* = 0.009). The anxiety/depression dimension at intake was also statistically significant with change in index (*p* = 0.002) and total sum scores (*p* = 0.022). Of the community exercise participants that reported mild-extreme problems on the anxiety/depression dimension at intake, 58 and 64% improved on the index score and total sum scores, respectively. Comparatively, participants that reported no problems on the anxiety/depression dimension at intake, 41 and 51% improved on the index and total sum scores, respectively.

In the physiotherapy program, only the anxiety/depression at intake was statistically significant with change in index (*p* < 0.001), VAS (*p* = 0.004), and total sum (*p* = 0.005) scores (Table 3). Of the physiotherapy patients that reported mild-extreme problems on the anxiety/depression dimension at intake, 83, 63, and 88% improved on the index, VAS, and total sum scores, respectively. Comparatively, participants that reported no problems on the anxiety/depression dimension at intake, 65, 49, and 75% improved on the index, VAS, and total sum scores, respectively (Table 3).

## Discussion

In this study, self-reported health based on the EQ-5D-5L was poor at intake, and despite important changes by the end of rehabilitation, health status remained generally lower than that of the general Alberta population [30]. Physiotherapy outcomes were positive with aggregate health status reaching general Alberta norms. Based on EQ-5D-5L index VAS, and total sum scores at end of care, physiotherapy had the highest proportions of patients improve (72, 55, and 81%, respectively) compared to the other service types, whereas pulmonary rehabilitation had the fewest patients improve (38, 46, and 47%, respectively). In each of the examined service types, a significant proportion of patients did not experience important changes in their health status based on the EQ-5D-5L, and some experienced a deterioration. However, for some, maintaining health status (i.e., no change) can be a successful outcome. Nonetheless, examining the reasons for lack of change or deterioration are important to identify those patients and better plan their care and follow-up. Further investigation in these areas could also be useful for decision making for other programs.

Few studies have examined routinely collected PROMs in the care of rehabilitation patients. However, the EQ-5D has been used to evaluate programs in similar clinical settings. Ernstsson et al. described how EQ-5D data is being used in 41 Swedish National Quality Registries of various patient populations (e.g., intervention assessment, health economics, quality indicators), including patients with conditions related to the musculoskeletal and nervous systems [31]. However, this report does not report any EQ-5D data and only provides a description of how EQ-5D results are presented and used at different levels of the Swedish health care system. Devlin et al. reported routinely collected pre- and post-operative PROMs data, including the EQ-5D-3L, from patients undergoing elective hip and cataract operations in the United Kingdom Department of Health, with the aim of demonstrating how EQ-5D data can be analysed and reported for the purposes of hospital performance evaluation [32]. Devlin et al. present the proportion of the sample reporting each of the levels ‘pre’ and ‘post’ on each of the dimensions, the PCHC and a health profile grid of patients’ index scores. A key finding was that many patients did not experience an improvement on EQ-5D scores following cataract surgery and the changes for many that did experience an improvement were small, prompting further investigation on the benefits of cataract surgery within the NHS. Both studies correspond to our study in that they demonstrate how EQ-5D data can be used to assess programs or interventions, and as quality indicators for following up on the quality of care.

Literature suggests that patients with pulmonary disease often experience impaired participation in activities of daily living in such a way that is out of proportion to lung function impairment [33, 34]. Targeted therapies to improve usual activities, mobility, and pain/discomfort may improve HRQL more than therapies that solely intend to improve patients’ lung function [33, 35, 36]. Moreover, based on results from this study, younger patients experienced “no change” in health status in greater proportion compared to older patients who experienced more “improvement”. Therefore, program interventions, based on age, could be explored. Likely, older patients have more severe or acute COPD and therefore have more “room” for improvement whereas younger patients may have higher unmet expectations of rehabilitation (e.g., being able to return to work). Additionally, community exercise program results highlight the need for further investigation as to why metropolitan region participants have better health status outcomes compared to rural regions, such as the differences in resources and services provided in each area.

In all programs, patients with problems with anxiety/depression at intake experienced more improvement in health status by end of care than those without problems. While this finding is the inverse of what we would expect, mental and physical health are highly correlated. Patients with problems in anxiety/depression may have more problems with their physical health and have more “room” for improvement compared to those without problems in anxiety/depression. The improvement of overall health status through rehabilitation intervention may also improve mental health. Several studies have found positive mental health outcomes through rehabilitation interventions [37–39]. Incorporating a mental health screen and referral, when appropriate, into the process of care could be considered in an effort to provide multidisciplinary comprehensive care for complex patient needs.

A multi-level framework for the various purposes of routinely collected PROMs in the health system is described by Al Sayah et al. [40] Micro-level use of PROMs data is to inform healthcare providers’ clinical practice and enhance patient engagement (e.g., screening/health monitoring, goal setting); meso-level use of PROMs data are aggregated from a group of patients within an organization and analyzed to monitor health outcomes or evaluate programs/services; macro-level use of PROMs data are aggregated to evaluate the performance of individual or organizational providers at the health system level by comparing health outcomes across jurisdictions/regions [40]. While the EQ-5D-5L can provide many uses for all levels described, all have different challenges. For instance, the EQ-5D-5L may not not provide significant programmatic value in comparison to condition specific PROMs, especially concerning therapeutic areas or aspects of health not captured by the EQ-5D-5L dimensions [6]. The EQ-5D-5L may not be sensitive enough to detect all the effects of rehabilitation services on HRQL. Another consideration is that the data represent just two ‘snapshots’ of patient-reported health status, at intake and end of rehabilitation. Therefore, the extent to which patient health may have deteriorated in the absence of rehabilitation services remains unknown [32]. Moreover, some of the deterioration in health observed between the two periods may be due to worsened general health due to aging or complications of co-morbidities [32]. Other challenges of successful PROMs implementation have been documented in the literature [9, 11, 41, 42]. However, the use of EQ-5D-5L or other PROMs data can illuminate issues for further investigation in improving rehabilitation services.

Our study has strengths, including robust sampling of real-world patients, however, it also has limitations. Foremost, we recognize this design does not provide a rigorous assessment of the effectiveness of community rehabilitation. There may have been a selection bias and non-response bias in the sample. Individuals with cognitive issues were not invited to complete routine PROMs. There could also be a differential loss to follow up of those who only completed an intake survey. The EQ-5D-5L has also not yet been implemented across all Alberta community rehabilitation sites, therefore the sample is not fully representative. Additionally, observed changes in health status may have been affected by reversion to the mean rather than natural progression of disease or condition. Conversely, the generic nature of the EQ-5D-5L may not have been able to capture specific aspects or changes of patients’ conditions. A limitation of our bivariate analyses was that compared to those who reported no problems on the EQ-5D-5L dimensions at intake, those who reported problems had more ‘room’ to change by the end of rehabilitation, therefore, overestimating the effect. And perhaps most importantly, it was not possible to link EQ-5D-5L data to any other patient data, which limited the analysis and interpretation of findings. PROMs are intended to be used alongside other outcome measures and not in isolation of other patient parameters. Similar PROMs explorations in evaluating healthcare services should be accompanied by examining other patient data. Despite these limitations, we hope the reporting of the use of EQ-5D-5L data can inform future developments in the routine use of PROMs in community rehabilitation settings.

## Conclusions

Routine collection of PROMs in the AHS Community Rehabilitation Program is intended to inform decisions at various levels within the system. AHS rehabilitation healthcare teams are committed to becoming data informed as they make improvements and inform future program or service changes [6]. Commitment to development of analytical and reporting methods of PROMs is key in health system management, with far reaching implications at all levels of healthcare. The implementation of the EQ-5D-5L as part of routine data collection in community rehabilitation, can increase the quality improvement culture with the clinical teams collecting and analyzing data. The results of this study are meant to inform the meso (i.e., program/service) level by describing the characteristics and health status of patients accessing community rehabilitation, as well as the predictors of change in health status, which will help direct future program growth and service changes.

## Data Availability

The data that supports the findings of this study are available from the University of Alberta. Restrictions apply to the availability of these data, which were used under license for the current study, and so are not publicly available. Data are however available from the corresponding author upon reasonable request and with permission of the corresponding author.

## Abbreviations

AHS: Alberta Health Services
VAS: Visual Analogue Scale
HRQL: health-related quality of life
MID: minimally important difference
PCHC: Pareto Classification of Health Change
PROMs: patient-reported outcome measures
R-MoC: Rehabilitation Model of Care
